# Chlamydia trachomatis-Induced Fitz-Hugh-Curtis Syndrome Presenting as Inspiratory Dyspnea

**DOI:** 10.7759/cureus.8566

**Published:** 2020-06-11

**Authors:** Shiva Malaty, Evan T Holdsworth

**Affiliations:** 1 Internal Medicine, HonorHealth Medical Center, Scottsdale, USA

**Keywords:** right upper quadrant pain, fitz hugh curtis syndrome, inspiratory dyspnea

## Abstract

Fitz-Hugh-Curtis syndrome (FHCS) is a rare complication of pelvic inflammatory disease. We report a case of a 21-year-old African American female presenting with inspiratory dyspnea and right upper quadrant pain found to be secondary to FHCS. The patient received antibiotic therapy, as well as testing of her sexual partners. This case demonstrates the importance of avoidance of biases such as anchoring, resulting in proper treatment and management of a rare disease entity.

## Introduction

Fitz-Hugh-Curtis syndrome (FHCS) involves inflammation of the liver capsule leading to the creation of adhesions from the anterior hepatic surface to the abdominal wall [1]. In addition to the low prevalence of this disease, the symptomatology of this condition, including right upper quadrant pain, nausea, and fever often mimics hepatobiliary pathologies such as cholecystitis, choledocholithiasis, and cholangitis, resulting in a nuanced clinical decision-making process. Therefore in order to identify this disease entity it is imperative to avoid medical decision-making biases such as anchoring. Proper identification and treatment of this syndrome may result in avoidance of any further medical complications such as infertility [2].

## Case presentation

**Figure 1:**
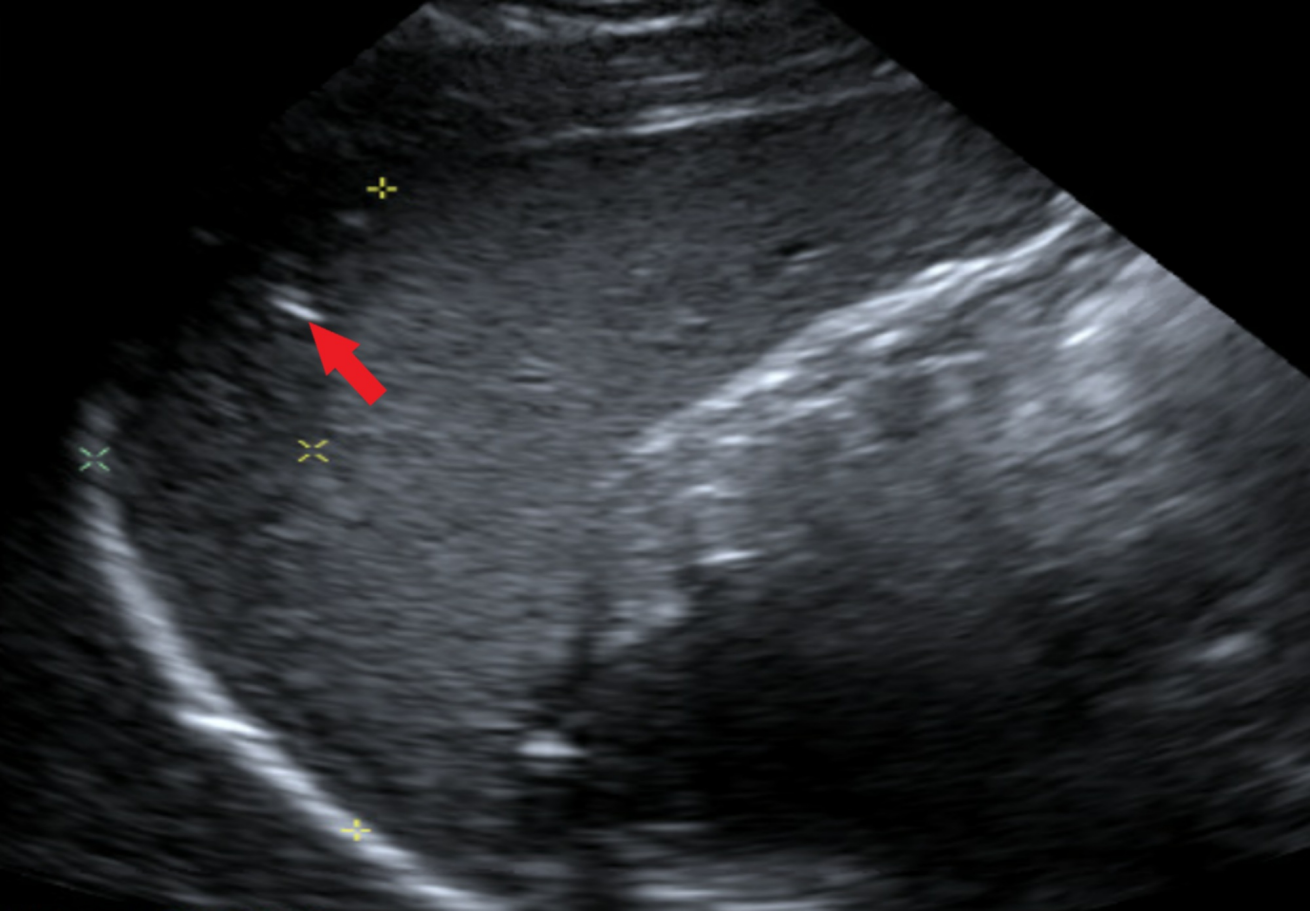
Complete abdominal ultrasound (US): Crescentic hypoechoic material around the edge of the spleen, underlying the dome of the left hemidiaphragm.

**Figure 2:**
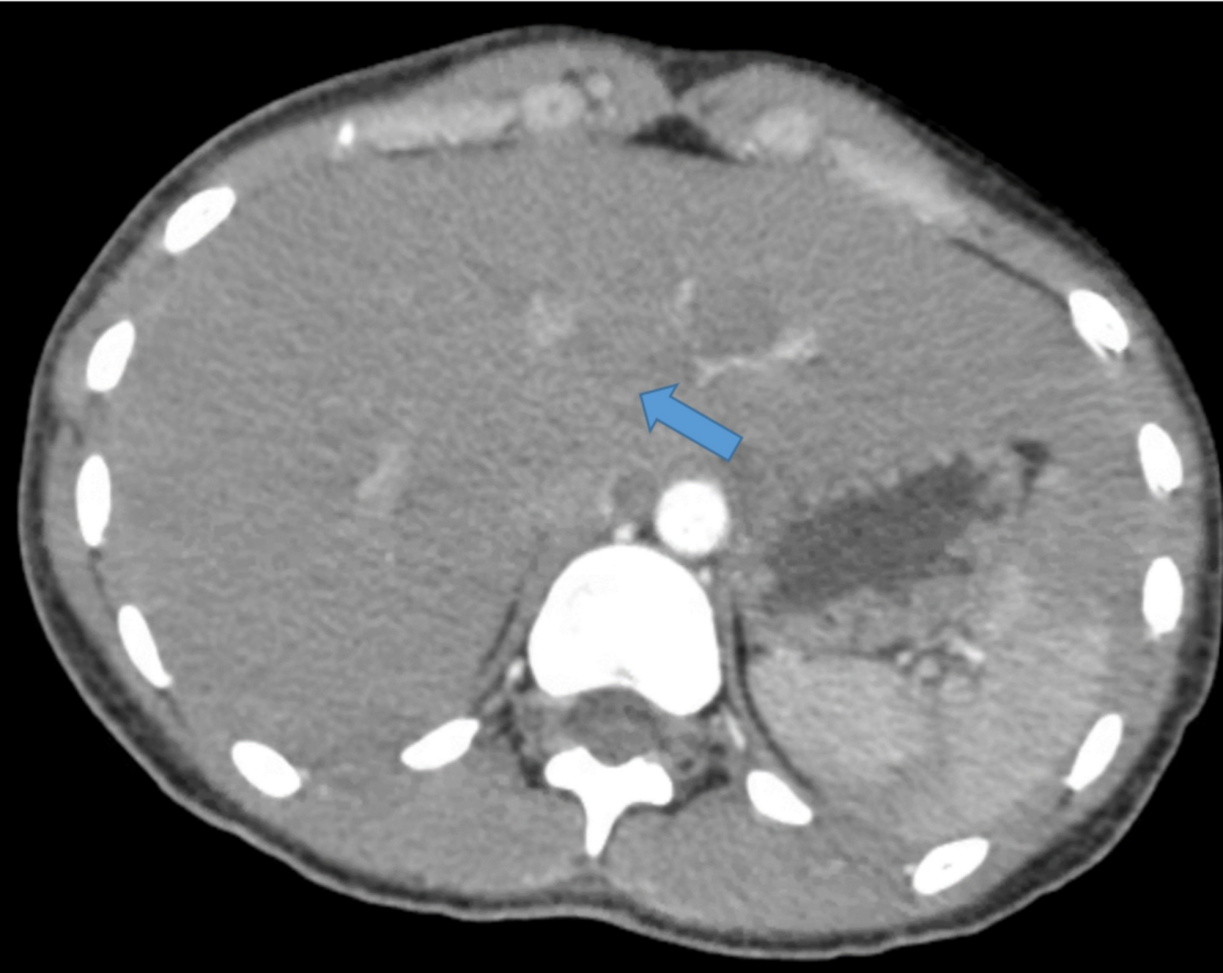
Computed tomography angiography (CTA) chest: Hepatomegaly, with generous left lobe wrapping laterally around the spleen. Mild upper abdominal ascites particularly in the perihepatic region.

We present the case of a 21-year-old African American female with past medical history significant only for peptic ulcer disease who presented with a chief complaint of shortness of breath on inspiration and associated right upper quadrant abdominal pain. Initial labs in the emergency department included a negative pregnancy test, elevated d-dimer, and an equivocal urinary analysis. On admission, physical exam demonstrated exquisite tenderness to light and deep palpation over the right upper quadrant without guarding and a negative Murphy’s sign. The physical exam was otherwise unremarkable. Empiric IV antibiotic therapy with metronidazole and rocephin was initiated. Upon initial history-taking, the patient denied any history of sexually transmitted diseases, but did report high-risk sexual behavior, prompting additional testing. HIV screen, Chlamydia trachomatis/Neisseria gonorrhea polymerase chain reaction (PCR), and rapid plasma reagin (RPR) were obtained. Figure 1 demonstrates the ultrasound of abdomen showed crescentic hypoechoic material around the edge of the spleen, underlying the dome of the left hemidiaphragm. Following symptoms of dyspnea and an elevated d-dimer, computed tomography angiography (CTA) of chest was obtained to assess for possible pulmonary embolism. Figure 2 demonstrates this imaging, which was significant only for hepatomegaly with generous left lobe wrapping laterally around the spleen, and mild upper abdominal ascites, particularly in the perihepatic region. In the setting of hepatomegaly and elevated d-dimer, concern for splanchnic vein thrombosis was raised, prompting an abdominal Doppler ultrasound study; hepatic vasculature was benign. PCR yielded a positive result for Chlamydia trachomatis, and was negative for Neisseria gonorrhea. HIV screen was nonreactive. After discussion of results with the patient, she admitted history of prior gonorrheal infection without completion of total antibiotic course. Given patient's PCR results, antibiotic regimen was changed to doxycycline. Clinical history, lab results, and imaging were consistent with a diagnosis of Fitz-Hugh-Curtis syndrome. The patient was discharged home with a 14-day course of doxycycline. The patient followed up one-week post-discharge with her pneumocystis carinii pneumonia (PCP) and showed significant symptomatic improvement.

## Discussion

Fitz-Hugh-Curtis syndrome (FHCS) is known as the great mimicker of acute cholecystitis; therefore, often times this diagnosis may be missed. In patients with pelvic inflammatory disease (PID), the total incidence of Fits-Hugh-Curtis syndrome has been reported to be around 4 to 27 percent [2]. The pathophysiology of this disease involves the spread of microorganisms in either hematogenous, lymphatic, or through spontaneous ascending infection [1]. There are many organisms found to be associated with PID, the most common causative pathogen of FHCS is found to be Chlamydia trachomatis [3].

Visualization of the adhesions with laparoscopy is the gold standard imaging for diagnosis [4]. However, given the invasive nature of these procedures a presumptive diagnosis is often made. Imaging modalities such as ultrasound and CT imaging play a role in ruling out other differentials. Additionally, patients with FHCS may have findings of capsular enhancement as well as inflammatory standing and fluid around the perihepatic region [5].

Treatment of FHCS involves targeting the causative organism with treatment dosing and length that would be appropriate for pelvic inflammatory disease. In this case, a fourteen-day course of doxycycline resulted in treatment as well as alleviation of patient’s symptoms. In addition to antibiotic therapy for the patient, proper testing and possible treatment of sexual partners should be done. If symptoms persist even after antibiotic therapy, then patient should undergo possible surgical evaluation [5]. Appropriate and prompt treatment aids in reduction of long-term complications including chronic pain, infertility, and small bowel obstruction.

## Conclusions

This case exhibits the importance of both thorough history-taking and examination as well as avoidance of anchoring and confirmation bias. Choosing to pursue the suspicion of a predetermined diagnosis typical of the right upper quadrant and neglecting to take a detailed social history may have resulted in misdiagnosis and mismanagement of this patient. By avoiding these biases, this patient, along with her sexual partners received proper medical management, potentially mitigating further disease transmission.
